# Real-Time Smart-Digital Stethoscope System for Heart Diseases Monitoring

**DOI:** 10.3390/s19122781

**Published:** 2019-06-20

**Authors:** Muhammad E.H. Chowdhury, Amith Khandakar, Khawla Alzoubi, Samar Mansoor, Anas M. Tahir, Mamun Bin Ibne Reaz, Nasser Al-Emadi

**Affiliations:** 1 Department of Electrical Engineering, College of Engineering, Qatar University, Doha 2713, Qatar; amitk@qu.edu.qa (A.K.); kalzoubi@qu.edu.qa (K.A.); sm1204406@student.qu.edu.qa (S.M.); a.tahir@qu.edu.qa (A.M.T.); alemadin@qu.edu.qa (N.A.-E.); 2 Department of Electrical, Electronic & Systems Engineering, Universiti Kebangsaan Malaysia, Bangi, Selangor 43600, Malaysia; mamun@ukm.edu.my

**Keywords:** digital stethoscope, heart diseases, heart sound, machine learning, Mel frequency cepstral coefficients (MFCC) features

## Abstract

One of the major causes of death all over the world is heart disease or cardiac dysfunction. These diseases could be identified easily with the variations in the sound produced due to the heart activity. These sophisticated auscultations need important clinical experience and concentrated listening skills. Therefore, there is an unmet need for a portable system for the early detection of cardiac illnesses. This paper proposes a prototype model of a smart digital-stethoscope system to monitor patient’s heart sounds and diagnose any abnormality in a real-time manner. This system consists of two subsystems that communicate wirelessly using Bluetooth low energy technology: A portable digital stethoscope subsystem, and a computer-based decision-making subsystem. The portable subsystem captures the heart sounds of the patient, filters and digitizes, and sends the captured heart sounds to a personal computer wirelessly to visualize the heart sounds and for further processing to make a decision if the heart sounds are normal or abnormal. Twenty-seven t-domain, f-domain, and Mel frequency cepstral coefficients (MFCC) features were used to train a public database to identify the best-performing algorithm for classifying abnormal and normal heart sound (HS). The hyper parameter optimization, along with and without a feature reduction method, was tested to improve accuracy. The cost-adjusted optimized ensemble algorithm can produce 97% and 88% accuracy of classifying abnormal and normal HS, respectively.

## 1. Introduction

Cardiovascular disease is one of the principal causes of human death in all over the world. Based on the American College of Cardiology, in 2008, over 616,000 persons died of heart disease, which caused almost 25% of deaths in the US, i.e., one in every four deaths [1]. In addition, a study from the British Heart Foundation stated that heart and circulatory system diseases were the second most common cause of death in the UK in 2014, with about 155,000 deaths. In 2014, cardiovascular disease (CVD) caused 27% of all deaths and cancers caused 29% [2]. Moreover, CVD is also a major cause of mortality in Australia, with 45,392 deaths (almost 30% of all deaths) attributed to CVD in 2015. CVD kills one person in Australia every quarter of an hour [3]. The last study that this was taken into consideration was in the “European CVD Statistics 2017 edition” article [4]. CVD is accountable for more than 3.9 million (45%) death in a year, where 1.8 million are males (40%) and 2.1 million are females (49%). The electrocardiogram (ECG), being the most popular, inexpensive, non-invasive, and intuitive method of diagnosing heart-related issues, has its limitation when it comes to detecting structural abnormalities and defects in heart valves due to heart murmurs [5]. Other technologies such as magnetic resonance imaging (MRI), which uses radio waves and magnets, are even capable of capturing moving images of the heart and major blood vessels [6]. Echocardiogram (echo) can also provide detailed anatomy of the human heart using the rebounding wave’s principle. Another known technology, which uses X-ray imaging, is the computed tomography (CT) scan of the heart. Recent advancement in 3D and 4D heart model reconstruction using MRI and CT helps to visualize more than a 2D image [7,8]. The major limitation of the above technologies is the use of complex machines, which are only affordable by large hospitals, and the World Health Organization (WHO) reports [9] that the majority of cardiovascular disease are suffered by low- and middle-income countries. In such countries, relying on the above technologies for diagnosing cardiovascular diseases could be unaffordable by the majority of the people in low-income countries for detecting the CVD in advance.

Understanding the characteristics of the heart sound (HS), also known as heart auscultation, has been one of the most primitive and popular methods of detecting early cardiac illnesses with the help of abnormal heart sounds. Phonocardiogram (PCG), also known as heart sound (HS), is a graph of the HS recording with the help of an equipment called as phonocardiograph [10]. There are three major limitations of the auscultation of the heart: Firstly, requirement of the device to be extremely sensitive as the sounds are of very low amplitude. Secondly, the low-amplitude HS signal can be easily corrupted by noise leading to faulty diagnosis. Finally, the reliability of the auscultation technique mainly depends on the skill, expertise, and capability of hearing of the doctor. Overcoming these limitations leads to motivation for work in this field.

**Figure 1:**
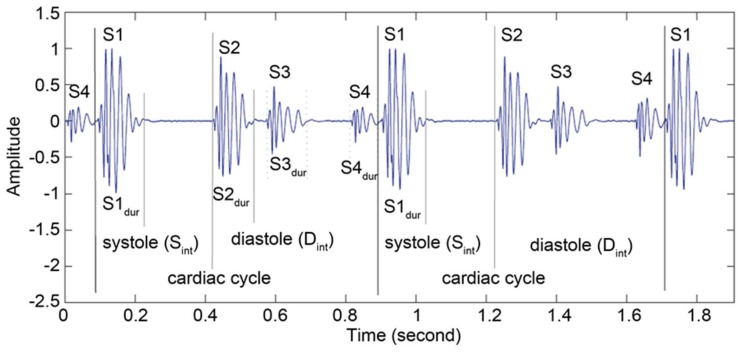
Different heart sounds.

The beating of the heart and the resulting flow of blood through the heart produces HSs. It is closure of heart valves that produces the normal heart sounds: Mitral and tricuspid valve closure produces the first heart sound (“S1”), and aortic and pulmonic valve closure produces the second heart sound (“S2”) (Figure 1). Heart valve opening does not normally produce a sound. Also, flow of blood from one cardiac structure to another is usually laminar and, therefore, silent under normal conditions. Problems in either heart valves or the heart muscles or both result in abnormal heart sounds and murmurs. The third HS (S3) (Figure 1) is normally caused by sudden reduction of blood supply from the left atrium to ventricle. In children and adults (35–40 years), this is normal. However, in other age groups and especially in the age group more than 40 years, it is abnormal and can be related to dysfunction or can be related to overloading of ventricles volume [11]. The fourth heart sound (S4) (Figure 1) can be related to failure of heart in the diastolic period. These heart sounds can be further characterized as the frequency of S1 as it is smaller than S2. The low-pitched sounds S3 and S4 occur 0.1 to 0.2 s after S2 and about 0.07 to 0.1 s before S1. There can be other HSs and heart murmurs that can be accounted for other cardiovascular issues [11].

The heart sounds S1 and S2 are high-pitched sound and heard well from the diaphragm of the stethoscope. The normal heart sounds S1 and S2 have frequency ranges of 50–60 Hz and 80–90 Hz, respectively [11]. S3 can be heard during the rush of blood entry to the ventricle from atrium and is normally a pre-diastolic low-pitched sound. When a failure of the heart is detected, S3 can be referred to as a bad extrapolative symptom. S3 has a bandwidth of 20–30 Hz [11]. The fourth heart sound (S4) happens at the end of diastole, which is a low-pitched sound and can be well-characterized by the bell of the stethoscope. S4 is not noticeable during atrial fibrillation or flutter [11]. The abnormal S4 has a frequency range below 20 Hz [11].

With the recent advancement in electronic technology, the digital stethoscope is gaining popularity day-by-day [12]. There are different types of digital stethoscopes [13,14,15,16] that have been industrialized to replace its analog counterpart. Electronic stethoscopes can provide better sound quality with variable amplification, minimize interference noise, and provide data for visualization and storage. Electronics stethoscopes still come equipped with connecting cables between the chest-piece and the head-piece, with the chest-piece having a wireless module to transmit the signal to receivers such as phone, digital audio recorder, or computers for recording and listening to the sounds [17].

Exploring heart illnesses using intelligent classification of HS/PCG is a highly motivated research topic. Research work with detailed reviews and state-of-the art techniques of PCG processing is stated in [12,18]. Research work explaining, in detail, the abnormalities with respective PCG characteristics is stated in [12,19]. The history of the evolution of using PCG for diagnosis is stated in [20], whereas the signal processing steps involved are discussed in [21]. Recent trends in machine learning techniques (automatic classification) and the feature selection for it is stated in [22] and [23]. Similar to the problems in machine learning techniques, different techniques of automatic segmentation, extraction of relevant features, feature reduction, and state-of-the art algorithms for classification have been discussed in several hundred articles in the literature.

Despite being audible, S1 and S2 have their amplitude vary and sometimes become very weak and could not be heard due to abnormalities. S1 and S2 do not have fixed frequencies but are within varied bands of frequencies in different cardiac periods. These limitations of the heart sound signal segmentation led researchers to develop a rather unique approach, which can be found in [11,18,24,25,26,27].

The features needed for PCG classification are of different types: (i) Time domain (t-domain), (ii) frequency domain (f-domain), (iii) time–frequency domain (t,f-domain), (iv) wavelet, and (v) Mel frequency cepstral coefficients (MFCC) features [23]. Several t-domain features were used by different groups for the PhysioNet-2016 challenge [28]. There are several f-domain features that were used for PCG classification, since the normal and abnormal HS have clear distinction in the frequency domain [29].

Time–frequency domain representation is well known to provide very useful information for sound wave classification. MFCC is the most commonly used time–frequency feature in the domain of automatic sound wave classification [30]. This feature has been widely used in many studies of automatic classification of sound signals. A large number of automatic PCG classification studies use wavelet-based features as a time–frequency representation as wavelets have certain advantages in terms of resolutions over short time Fourier transform STFT, which is used in MFCC [31].

Many studies using time–frequency domain features of PCG signal have used deep learning techniques for classification with reasonable accuracy [32,33]. In the PhysioNet-2016 challenge [28], most of the top teams used these features [28,29,30,34]. It is stated that, amongst the submissions of the various teams in the challenge, the highest score of the competition for sensitivity, specificity, and overall scores were 94.24%, 77.81%, and 86.02%, respectively [28] and therefore, there still is scope for improvement. In all the works stated above, numerous automatic machine learning algorithms were tested and reported for different public databases. However, the combination of t-domain, f-domain, and (t,f)-domain with a reasonable number of features, which can be applicable for real-time classification with high accuracy for classification, have not been presented yet in any of the recent works. Moreover, to the best of our knowledge, none of them have employed hardware solution for acquisition of PCG signal and real-time machine learning to classify the heart sounds into normal and abnormal. This will help the home users to initially evaluate their disease at home without visiting hospital frequently and, if an abnormality is observed, can go for clinical expertise to diagnose a medical condition based on the heart sounds.

This article is organized into five sections. In the first section, the review of different heart sounds and recent works for their classification along with the motivations of this work are summarized. The experimental details and methods are presented in Section 2 with subsections along with different hardware and software studies. We explain the method of analysis as pre-processing steps of system evacuation in Section 3, followed by results and discussion in Section 4. Section 5 concludes the work with future directions.

## 2. Experiment Details and Methods

**Figure 2:**
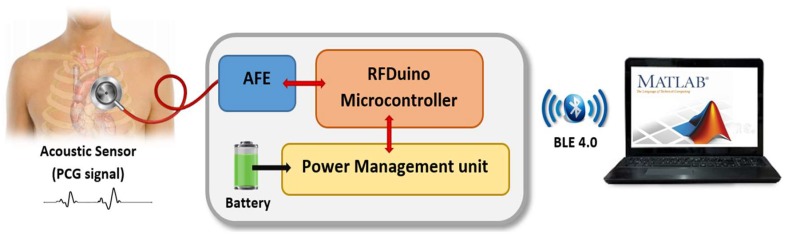
Overall system block diagram.

The prototype system consists of two subsystems that communicate wirelessly using Bluetooth low-energy (BLE) technology: Sensor subsystem, and an intelligent detection subsystem as shown in Figure 2. The acoustic sensor firstly acquired the heart sounds signal and fed it to analog-font-end (AFE), where it has a pre-amplification and filtering of the heart sounds signal. After that, the signal is converted by ADC in the RFduino microcontroller and transmitted wirelessly into a personal computer (PC) where the signal will be processed and classified using MATLAB.

**Figure 3:**
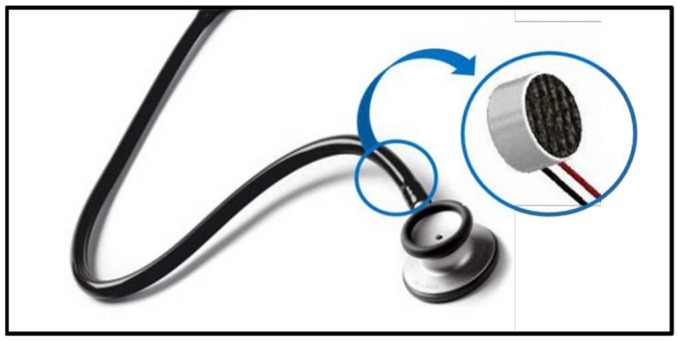
Customized acoustic sensor.

**Figure 4:**
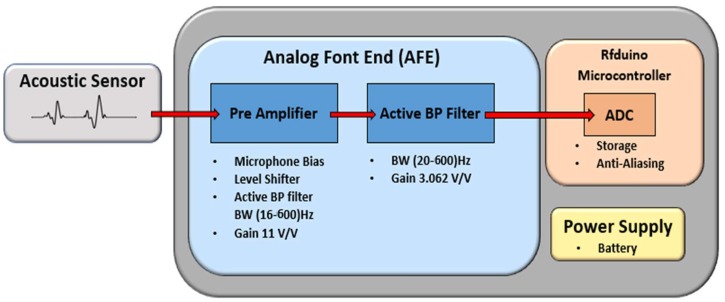
Detailed block diagram of the sensor subsystem.

A real-time heart sound signal acquisition, amplification, filtering, digitization, and wireless transmission are accomplished by the sensor sub-system. A custom sensor was designed and implemented using a traditional stethoscope chest piece to amplify the heart sound waveform. A small microphone sensor with bandwidth of 20–600 Hz is selected to perform the conversion of the heart sound to electrical signal. The microphone was placed in the rubber tubing very close to the chest piece as shown in Figure 3. This also includes analogue front end (AFE), and RFduino microcontroller with embedded Bluetooth low-energy (BLE) module. The custom-built stethoscope acquires the acoustic signal, amplifies and filters it through AFE, and then digitizes and transmit the raw data to the decision-making subsystem (Figure 4). The AFE is required to maintain a high signal-to-noise ratio (SNR), high common mode rejection, and less baseline drift and saturation problems. The pre-amplifier circuit takes the very weak heart sound signals from a microphone and amplifies it to the suitable level.

**Figure 5:**
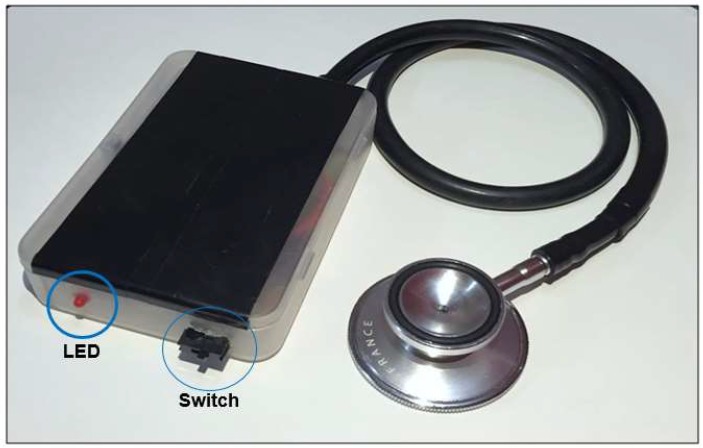
Overall implemented phonocardiogram (PCG) signal acquisition system.

The ARM Cortex M0 is the core of RFduino microcontroller and it has a built-in Bluetooth 4.0 low-energy module. RFduino uses Arduino IDE as user interface program, which allows testing and running of pre-written sketches and takes advantage of the existing libraries. RFduino has 10-bit analog-to-digital (ADC) module, which is capable of acquiring the acoustic signal at 500 Hz sampling rate with the resolution of 2.93 mV. Moreover, the dimension, low-power consuming feature, 3.0 V operating voltag,e, and built-in BLE module made RFduino an excellent choice for this application. The sensor subsystem is powered through a Li-ion battery that is connected directly through a PowerCell board. The power management module (PMM) is a boost converter (to 3.3 V and 5 V) and micro-USB charger in one. The boost converter is based on the TPS61200 from Texas Instrumentation (TI) and has solder jumper selectable 5 V and 3.3 V output, and an under-voltage protection of 2.6 V. The module can be charged by mobile charger using an on-board micro-usb connector and is capable of delivering 3.3 V or 5 V. The PMM is configured to provide 3.3 V output to the RFduino, and the AFE module. The final stethoscope containing the circuitry of the system designed using OR-Cad software is shown in Figure 5.

The intelligent abnormal heart sound and warning subsystem is the brain of the whole system and plays a major role in the system operation. It is made up of three blocks: Bluetooth module, data acquisition and logging, and classification. This module detects the event of abnormal heart sound in real-time manner depending on the acquired acoustic signals and the trained machine learning model. To acquire wireless PCG signal over Bluetooth, RFduino module with USB shield was used to provide wireless interface of intelligent system with the sensor system. The heart sound was received in the computer over BLE interface from RFduino. The classification algorithm was not implemented in the stethoscope itself and this task was not feasible yet with the low computational capability of the chosen microcontroller for data digitization and transmission, however the classification was done online in the PC. Two phases of implementations are described in this work: (a) Matlab-based implementation to find the best-performing optimized algorithm, and (b) Python-based real-time signal processing and classification.

We have conducted a series of experimental tests to conduct the hardware and software evaluation.

### 2.1. Evaluation of the Signal Fidelity of Prototype Sensor Subsystem

**Figure 6:**
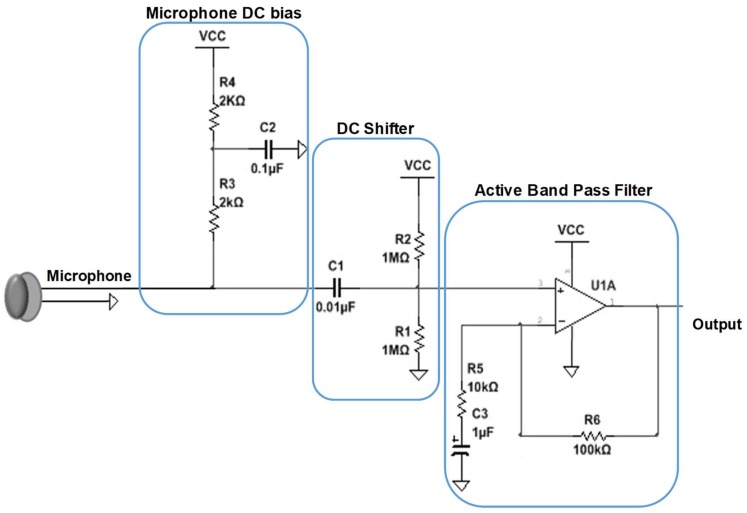
Schematic of the pre-amplifier of the sensor system.

The quality of the heart sound signal acquired by the prototype model is compared with the commercial 3M Littmann Classic III Monitoring Stethoscope (3M Health Care, Conway Ave., St. Paul, MN, USA). The analog front end of the prototype system is mainly made up of the pre-amplifier and band pass filter. The pre-amplifier provides amplification with gain of 11 *v*/*v*. Moreover, it provides DC shifting controlled by the resistors R2 and R1 to remove the negative components of the signal (Figure 6), which is necessary for the analog-to-digital converter (ADC). The resistors R4 and R3 DC-bias are the microphone input (Figure 6).

**Figure 7:**
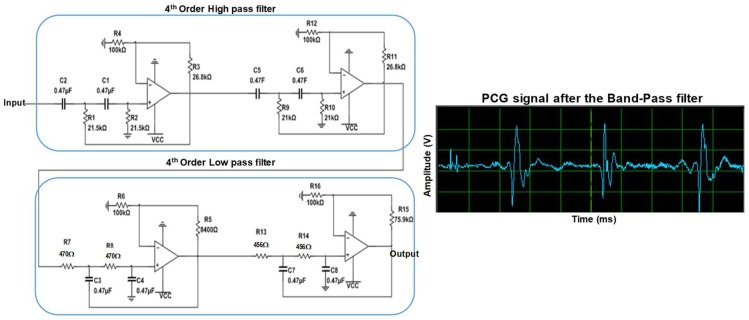
Schematic of fourth-order Bessel band pass filter.

In addition, the design has a first-order band pass filter of 20–600 Hz cutoff frequencies used to provide extra filtering to the signal. The Bessel filter was selected to perform the filtering of the HS signal as it has a linear phase shift that is necessary for audio signal filtering. The design consists of a fourth order high-pass filter (HPF) followed by a fourth-order low-pass filter (LPF). The bandwidth of the filter was chosen to be between 20 Hz to 600 Hz, which is sufficient to record a clean HS signal. The filter has a total gain of 3.06 *v*/*v* as shown in the following calculation below. The MCP604 quad operational amplifier (Figure 7) was used for designing the filter as it provides low-bias current, high-speed operation, and rail-to-rail output swing.

This HS signal was transmitted to PC over BLE interface and compared with the HS signal from the commercial 3M Littmann Classic III Monitoring Stethoscope.

### 2.2. Evaluation of the Reliability of the BLE Transmission System

RFduino uses Gazell (GZLL) BLE protocol to transmit data between the sensor subsystem and the decision-making subsystem. It is a proprietary packet radio protocol released by Nordic Semiconductor, which uses a star topology with one host and up to seven devices. In the GZLL protocol, the devices can communicate only with the host but cannot communicate directly with another device. The host can coordinate packets between devices.

In our system, RFduino attached to sensor module initiated the request for data transmission and RFduino in the PC-side was working as host was responsible for receiving the data packet. To ensure reliable data transmission to the host without missing any data packet, acknowledgement of data packet reception in BLE buffer was used. Moreover, to increase the sampling frequency of the RFduino to 2000 Hz in data acquisition, heart sound data were buffered in the RFduino before transmission and after every 20 ms buffered frame, 40 PCG samples were sent to host. This was to ensure low-power consumption of the wearable system while keeping high-frequency sampling for reliable heart sound signal acquisition. RFduino timer interrupt was used to ensure 0.5 ms interrupt-driven data acquisition for guaranteeing 2000 Hz sampling frequency.

**Figure 8:**
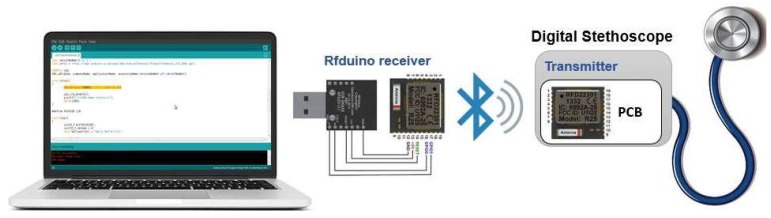
Communication between the two subsystems.

An experiment was conducted to check the performance of the wireless transmission system in transmitting the heart sound data over the wireless interface and evaluate the fidelity of the signal at 2000 Hz sampling frequency. Figure 8 is showing a schematic representation of the wireless communication.

### 2.3. Evaluation of Battery Life of the Sensor Subsystem

**Figure 9:**
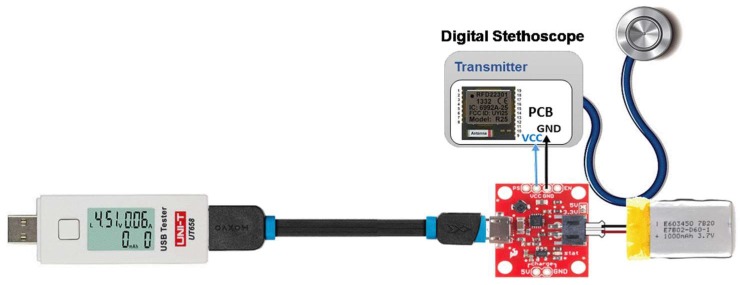
Evaluation of overall power consumption of the sensor module.

The Sparkfun power cell charger/Booster Module was selected as power management module (PMM) for the system. A lithium polymer battery (LiPo) of 3.7 V (300 mAH) was used with the module along with PMM, and is capable of delivering 3.3 V to the system. The battery life can be found using the following equation, where the factor 0.70 (https://www.digikey.com/en/resources/conversion-calculators/conversion-calculator-battery-life) then makes allowances for external factors that can affect the battery life. Figure 9 shows the experimental setup for measuring power consumption of the portable system.

(1) $$Battery\;Life=\frac{Battery\;Capacity\;\left(mAh\right)}{Load\;Current\;in\;mill\;amps}\;\times 0.70$$

### 2.4. Performance Evaluation of Machine Learning Abnormality Detection Algorithms

Normal and abnormal heart sound data from a public PhysioNet-2016 challenge database were used for training and testing of the machine learning algorithms in the Matlab environment to identify the best-performing algorithm and optimize the parameters of the best-performing algorithms to obtain the highest accuracy.

#### 2.4.1. Database Description

PhysioNet challenge 2016 dataset consists of five databases (A through E) containing a total of 3126 heart sound recordings, lasting from 5 s to just over 120 s. These HS data were recorded from clinical and nonclinical environment from both healthy and pathological patients (e.g., children and adults) from four different locations—aortic, pulmonic, tricuspid, and mitral areas. In both training and test sets, heart sound recordings were divided into two types: Normal and abnormal heart sound recordings. Both the training and test sets are unbalanced, i.e., the number of normal recordings does not equal that of abnormal recordings. The number of normal recordings is higher than abnormal recordings. The recordings last from several seconds to up to more than 100 s. All recordings have been resampled to 2000 Hz and have been provided as.wav format. Each recording contains only one PCG lead.

#### 2.4.2. Optimized Classification Model Selection

**Figure 10:**
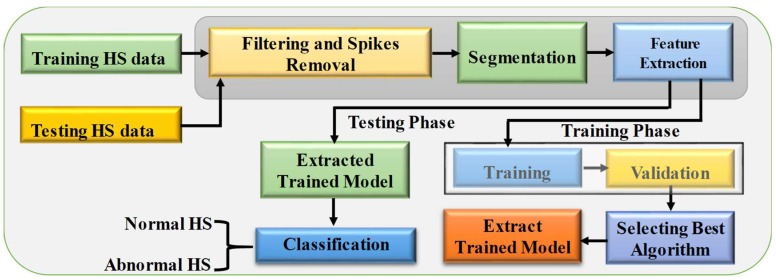
Blocks of the machine learning-based abnormality detection algorithm.

The classification model selection is shown (Figure 10) with work flow diagram of the signal pre-processing and application of machine learning algorithm. The HS data from the database were segmented to training and testing datasets. Signal pre-processing and automatic segmentation were accomplished using the signal processing toolbox, and training and classification of HS were accomplished by the statistics and machine learning toolbox in the Matlab 2018a. The pre-processing steps of the HS are summarized in the analysis section. Several time (t)-domain, frequency (f)-domain, and Mel frequency cepstral coefficients (MFCC) features were extracted from the segmented HS data. The training dataset underwent pre-processing steps before it was fed into the machine learning algorithms for training. The details of the pre-processing steps and detection algorithm will be discussed in the analysis section.

Twenty-two different ML algorithms (three decision tree, two discriminant analysis, six support vector machines (SVM), six k-nearest neighbor (KNN), and five ensembles classifiers) were trained with five-fold cross-validation using 27 features of the training dataset and the best-performing algorithm was identified. Feature reduction and hyper-parameter optimization was used to optimize the best-performing algorithm. Their validation accuracy, sensitivity, and specificity along with other performance metrics were evaluated. The trained model was used for calculating the performance evaluation matrix for the testing data in identifying the normal and abnormal HS.

### 2.5. Real-Time Classification of Heart Sound Signals

For the real-time implementation, heart sound data were buffered for 10 s and then the baseline drift was corrected, segmented to heart sound beats (one heart sound trace), and band-limited filtering in Python 3.5. A multi-threaded python script was written to acquire, buffer, real-time pre-process, and classify the heart sound data in the host computer. Signal pre-processing (such as real-time filtering and feature extraction) and segmentation were implemented in a PC using Numpy (v1.13.3), scikit-learn (v0.20), and Matplotlib (v3.0.2) libraries. The best-performing algorithm was then implemented in the PC for real-time classification using PyBrain (v0.31) and Scikit learn (v0.20) libraries. The decision of the real-time classifier is displayed in a graphical user interface (GUI) built on tkinter module. For real-time testing, PCG signals were acquired from six healthy (three males and three females, age range: 20–45 years, mean: 31.2 years, standard deviation: 9.6 years) and six patients with murmur (three males and three females, age range: 39–56 years, mean: 46.7 years, standard deviation: 6.4 years) with written informed consent from subjects and approval from local ethics committee.

## 3. Analysis

There are several pre-processing steps that were applied to the heart sound data before it can be used by the machine learning algorithm. During the training and testing period, these steps were done manually, and these were made automatic for real-time classification.

### 3.1. Pre-Processing Steps

The following pre-processing steps were carried out to filter the noises and spikes, and segment the HS data:

#### 3.1.1. Filtering and Spikes Removal

A sixth-order bandpass IIR filter with the lower cut-off frequency of 20 Hz, higher cutoff frequency of 600 Hz, and the sample rate of 2000 Hz was used to filter the HS data to remove any potential low-or high-frequency noise.

#### 3.1.2. Segmentation

**Figure 11:**
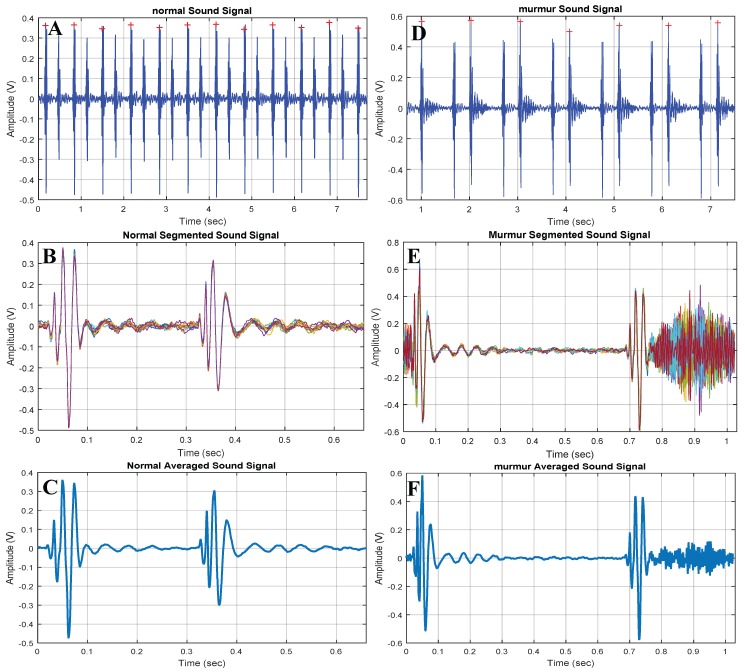
Normal and abnormal heart sounds (HS): (**A**,**D**) detection of peaks; (**B**,**E**) overlaid segments; (**C**,**F**) average of the segments.

Segmentation of the PCG signals into heart cycles or marking of cycle starting instances are very important to generate the epoch of interest for training, and testing of the machine learning algorithm. There is much literature and state-of-the-art tools publicly available for segmenting the HS data. Since the location of the HS acquisition place has significant influence on the noise contamination to the PCG signal; therefore, extraction of the heart cycle period reliably is a challenging task. However, when the PCG signal was recorded with electrocardiogram (ECG) signal, this segmentation process become comparatively easier as ECG R-peaks are more distinct than the PCG signal’s S1 and S2 peaks. In this work, we have automatically identified the S1 peaks, which are the most dominant peak of the PCG signal. PCG signal between one S1 peak to another S1 peak was used mainly as the heart cycle along with offset to capture the beginning of S1 signal and ending of S2 signal. Figure 11 shows how the normal and abnormal PCG signal of several seconds were segmented to heart cycle.

### 3.2. Feature Extraction

**Figure 12:**
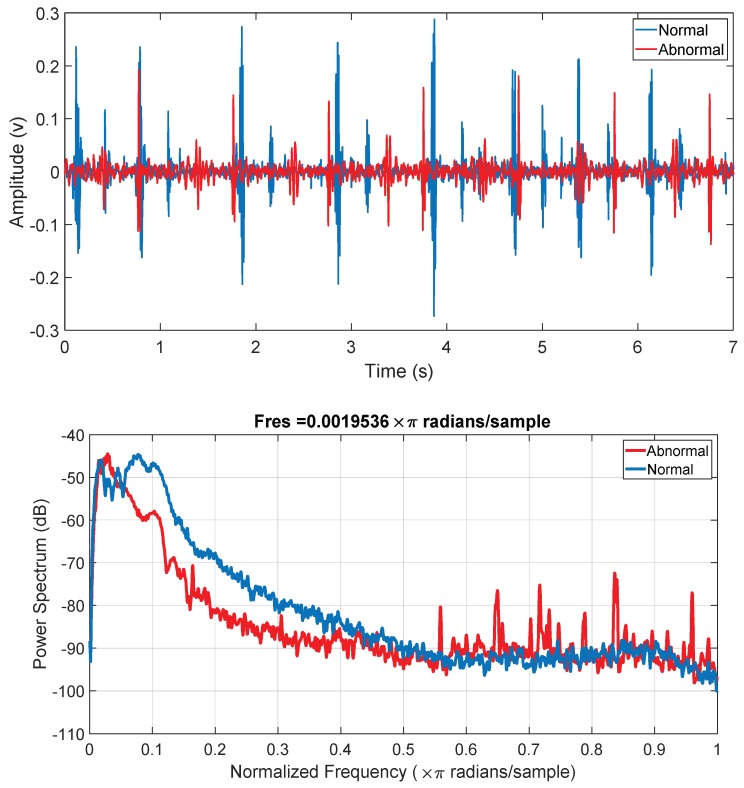
Time domain PCG trace and its power spectral density for normal and abnormal subjects.

The power spectral of the signal in Figure 12 shows that the power spectral density peaks appear at different frequencies for normal and abnormal PCG signals. Moreover, the power spectral density at higher frequency has no peak for normal PCG signal, whereas this is not the case for abnormal PCG signals and there are several peaks in between 300 Hz to 600 Hz. This reflects that the simple frequency domain feature can help significantly in classifying the PCG signals. However, more t-domain, f-domain, and Mel frequency cepstral coefficients (MFCC) provide insight on the signal while compensating for the noise or motion artefacts. Moreover, it has been shown in the literature that the MFCC features can contribute significantly in classifying the sound waves. Therefore, 27 features encompassing t-domain, f-domain, and MFCC features were extracted for each heart sound cycle (Table 1). The t-domain, f-domain and MFCC features used in this study are taken based on the previous works [28,29,30]. The t-domain features were: Mean value, median value, standard deviation, mean absolute deviation, signal 25th percentile, signal 75th percentile, signal interquartile range, skewness, kurtosis, and Shannon’s entropy; whereas the f-domain features were: Spectral entropy, maximum frequency in the power spectrum, signal magnitude at maximum frequency, and ratio of signal energy between maximum frequency range and overall signal. The rest of the features were MFCC features.

### 3.3. Classification

A large variety of machine learning (ML) algorithms can be used for classifying the HS signals into normal and abnormal. In the subsequent section, we will discuss the training and validation of different ML model, and testing of the pre-trained best performing model for classification of HS signals to detect abnormality in a real-time manner.

#### Performance Evaluation Matrix

The benchmark dataset was randomly partitioned into two subsets: (i) Training and validation set (80% of data), and (ii) testing set (20% of data). The ML model were trained for two classes: “normal” and “abnormal”. To compare the performance of several ML algorithms in classifying PCG signals, confusion matrices for each algorithm after 5-fold cross-validation were created and several standard statistical evaluation parameters were calculated to evaluate the performance of the algorithms:

True Positive Rate (TPR)/Recall/Sensitivity:

(2) $$Recall=\;\frac{TP}{TP+FN}$$

Specificity:

(3) $$Specificity=\;\frac{TN}{TN+FP}\;$$

False Positive Rate (FPR):

(4) $$FPR=1-Specificity=\frac{FP}{TN+FP}\;$$

Precision:

(5) $$Precision=\;\frac{TP}{TP+FP}$$

F-measure or score:

(6) $$F\;score=\frac{2*Recall*Precision}{Recall\;+\;Precision}\;$$

Accuracy:

(7) $$Accuracy\;\left(ACC\right)=\frac{TP+TN}{P+N}$$

Error:

*Error* = 1 − Accuracy (8)

(9) $$\mathrm{Matthews}\;\mathrm{correlation}\;\mathrm{coefficient},\;MCC=\frac{\left(TP*TN-FP*FN\right)}{\left((TP+FP\right)*P*N*\left(TN+FN\right){)}^{0.5}}\;$$

where TP is true positive, TN is true negative, FP is false positive, FN is false negative, P = TP+FN and N=FP+TN.

The above-mentioned parameters were estimated using 5-fold cross validation such that the training database was divided into five equal sets. Out of five sets, four sets were used for training while one set was used for testing. This process is repeated five times such that each set is tested once. The final results are obtained by averaging the results of all the iterations. The average of all the above-mentioned parameters were calculated. Performance evaluation of three different best-performing ML algorithms were calculated to identify best one in the testing phase.

### 3.4. Feature Reduction

Neighborhood component analysis (NCA) is a non-parametric and embedded method for selecting features with the goal of maximizing prediction accuracy of classification algorithms. The Statistics and Machine Learning Toolbox™ built-in functions can be used to perform NCA feature selection with regularization to learn feature weights for minimization of an objective function that measures the average leave-one-out classification loss over the training data. It was found that the most contributory features are 15 features out of the 27 features. These are kurtosis, maximum frequency value, and all of the MFCC features. The ML algorithms were trained again with the same training data subset with the reduced feature matrix to see whether this feature reduction can improve the classification accuracy by reducing over-fitting or not. Performance measures were calculated for the three best-performing algorithms to identify the best one for the testing data subset.

### 3.5. Hyperparameter Optimization of the Best-Performing Algorithm

Each of the trained machine learning algorithms were trained with some default parameters, which produce a particular validation accuracy; however, these algorithms can be tuned to optimize their hyperparameters. It would be a very tedious task to tune all the algorithms trained to check the validation accuracy. Therefore, the best-performing algorithms were optimized to calculate the performance measures. The accuracy and other performance measures were then calculated for the testing dataset (20% of the whole database).

### 3.6. Unequal Misclassification Costs

It is apparent from Table 2 that the number of abnormal and normal observations for both the training and testing datasets are unequal or the dataset is imbalanced. Moreover, misclassifying observations of abnormal class has more severe consequences than misclassifying observations of normal class. Since the classes are adequately represented in the training data but we have to treat them asymmetrically, the cost of classes were made different. Since we want to classify patients with normal and abnormal heart sounds, failure to identify an abnormal class (false negative) has far more severe consequences than misidentifying normal class as abnormal (false positive). We have assigned 10 times more cost to misidentifying abnormal HS as normal HS and low cost to misidentifying normal HS as abnormal HS. We have trained best-performing classification algorithm with unequal classification costs to increase accuracy for abnormal HS with higher accuracy by partially scarifying the accuracy of the normal HS classification. This partial reduction in the accuracy in normal HS classification should be acceptable, as this is only a screening test.

## 4. Results and Discussion

This section summarizes the results from the hardware experiments and algorithm performance evaluation studies.

### 4.1. Evaluation of the Signal Fidelity of Prototype Sensor Subsystem

**Figure 13:**
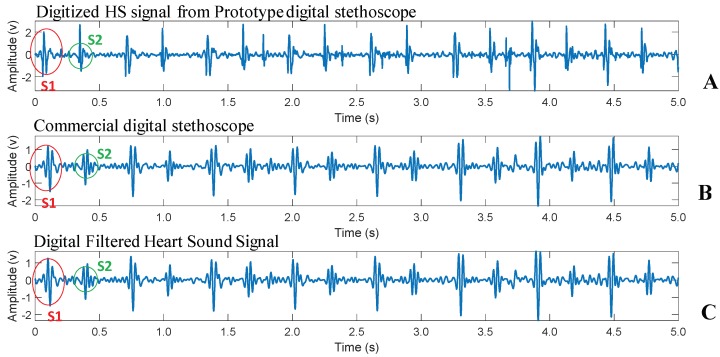
Comparison between the output PCG signal from prototype stethoscope (**A**), commercial stethoscope (**B**), and band-limited PCG signal prototype system (**C**).

A commercial 3M Littmann Classic III Monitoring Stethoscope was used as a reference to be compared with the designed digital stethoscope output. The Littmann Classic III digital stethoscope was set to bell mode that has a bandwidth of range 20–600 Hz to match the frequency range of the designed stethoscope. Both systems were setup to sample the captured heart sounds signal using sampling frequency equal to 2 KHz. It was important to change the digital stethoscope sampling frequency to 2 KHz for accurate comparison. Diaphragms (chest-piece) of the Littmann stethoscope and prototyped stethoscope were placed simultaneously on the chest of the subject under study. Figure 13 shows the heart sounds signal of the designed digital stethoscope and its digital filtered version and also shows the results of the commercial digital stethoscope from subject 1. Both signals were captured at the same time from the same subject (subject 1) while the chest-piece was placed on the chest (horizontally side-by-side) simultaneously. The prototype digital stethoscope signal shows clearly the same S1 and S2 components shown in the commercial one; however, it has more amplification than the commercial one in the original signal (Figure 13A) and closer to the commercial one in the digitally band-limited version (Figure 13C).

### 4.2. Evaluation of the Reliability of the BLE Transmission System

**Figure 14:**
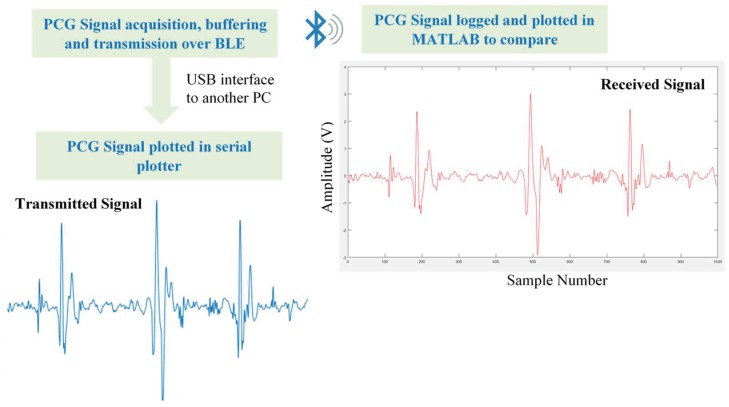
Test setup for Bluetooth low-energy (BLE) reliability evaluation along with transmitted and received PCG signal.

**Figure 15:**
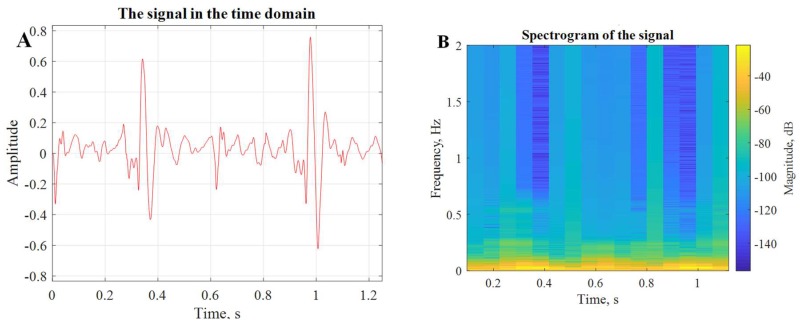
Representation of (**A**) time domain and (**B**) time-frequency domain analysis of the received signal.

The HS signals were transmitted over BLE to the decision-making subsystem as mentioned in the methodology to compare the transmission reliability. However, to evaluate the transmission reliability, a special testing arrangement was made (shown in Figure 14). In test-setup, the wearable system’s RFduino was connected to a PC using USB interface while sending PCG data packets to the host computer over BLE using GZLL protocol. The PCG signal over BLE was logged in the host computer and plotted using MATLAB to compare with the transmitted signal. It was compared packet by packet to identify any discrepancy of the received data (i.e., packet loss) during the transmission. It was observed that acknowledged and frame-based data transmission ensures the communication reliability and there was no packet loss observed in the transmission. The received signal was evaluated in time domain and time–frequency domain to observe any potential missing frequency components in the received PCG signal, as shown in Figure 15.

### 4.3. Evaluation of Battery Life of the Sensor Subsystem

The load current of the sensor circuit was found to be 1.18 mA, and the current when there was BLE transmission was 6 mA; thus, the total current consumption of the sensor circuit and BLE transmission would be 7.18 mA. The power consumption measurement was done utilizing the arrangement, as shown in Figure 9. The rating of the battery is 300 mAh and the battery life is (300 mAh/7.18 mA) × 0.7 ≈ 41 h; the system can be powered continuously for around 1.5 days from that battery if we consider that the BLE is continuously running even though, in practice, data were sent over BLE every 20 ms. However, the average current is 1.48 mA and battery life should be (300 mAh/1.48 mA) × 0.7 ≈ 142 h (i.e., 5.9 days). Therefore, overall battery life of the system will be several days.

### 4.4. Performance Eevaluation of Machine Learning Abnormality Detection Algorithm

Twenty-two different algorithms (three decision tree, two discriminant analysis, six support vector machines (SVM), six k-nearest neighbor (KNN), and five ensembles classifiers) were trained using 27 features of the training dataset (80% of the whole dataset). The validation accuracy and their corresponding performance measures are listed in Table 3.

It is obvious from the above table that the best validation accuracy was observed for “Fine Tree” classifier. Moreover, the accuracy of classifying normal is higher than abnormal and this is because of the imbalanced dataset as shown in Table 2. Therefore, we needed to check the potential over-fitting of the features. This could be dealt with by reducing the number of features used in the training process. Therefore, the training dataset was retrained with the reduced number of features (15) and the confusion matrix and evaluation measures were calculated. Table 4 summarizes the accuracy and other evaluation measures for identifying the best algorithm after feature reduction.

However, it is apparent from Table 4 that the overall accuracy was reduced, and classifying normal and abnormal were also both reduced even though the same algorithms were performing best in the classification after feature reduction. Therefore, it can be said that the features used for classification are optimized and cannot be reduced.

**Figure 16:**
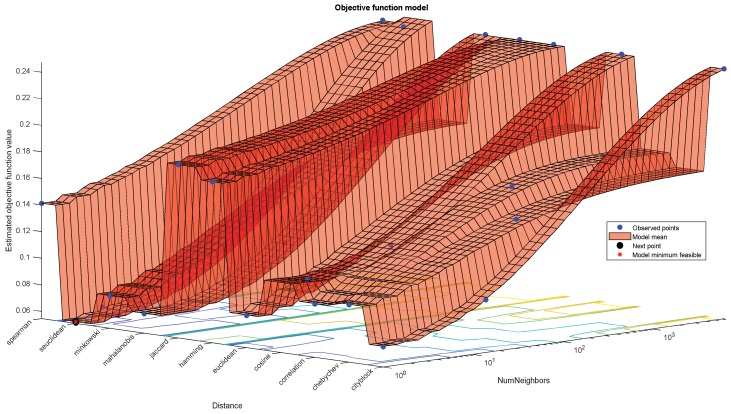
Optimization of hyperparameter for ensemble algorithm.

**Figure 17:**
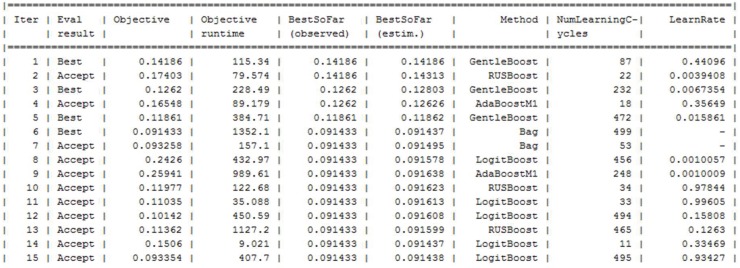
Number of evaluations to reach minimum objective.

To improve the performance of the best-performing algorithms by optimizing the hyperparameters of the algorithms, it was observed that the performance of the ensemble algorithm can be improved. Two important parameters were optimized for the ensemble algorithms: “Distance” and “Number of neighbors”. Figure 16 shows the optimization of two parameters and Figure 17 shows the number of required iterations to reach the objective.

**Figure 18:**
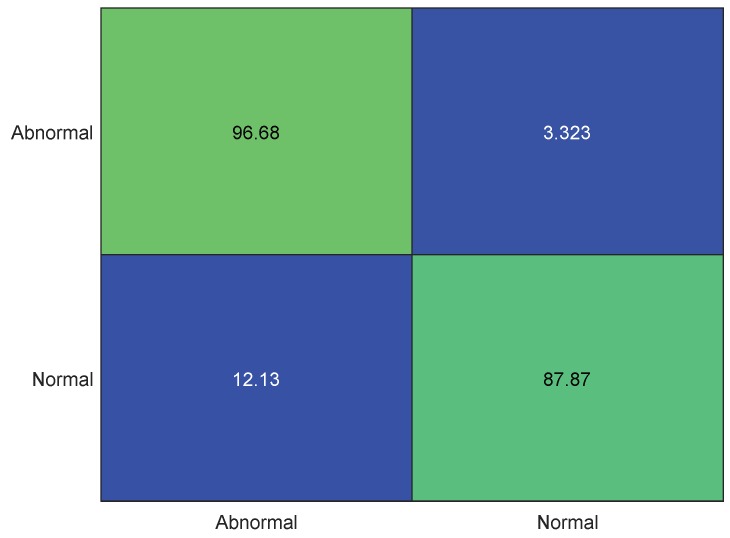
Confusion matrix for hyperparameter optimized ensemble algorithm for test dataset.

The best-performing and hyperparameter-optimized algorithm was retrained with cost adjustment to make sure that the “abnormal HS” will be classified with more confidence than the “normal HS”. The result of this asymmetric cost adjustment is shown in Figure 17. It is evident that the classification accuracy of abnormal HS became approximately 97% while that is for normal HS is approximately 88% (Figure 18). The team with the highest score of the PhysioNet-2016 challenge competition reported sensitivity, specificity, and overall scores of 94.24%, 77.81%, and 86.02%, respectively [28]; however, in this work, an overall sensitivity, specificity, and accuracy of 96.32%, 89.34%, and 94.63%, respectively were attained. It therefore can be observed that the trained model can much more reliably classify abnormal HS sound with 96.68% accuracy and normal HS with 87.87% for the testing dataset, as shown in Figure 18.

### 4.5. Real-Time Classification of Heart Sound Signals

**Figure 19:**
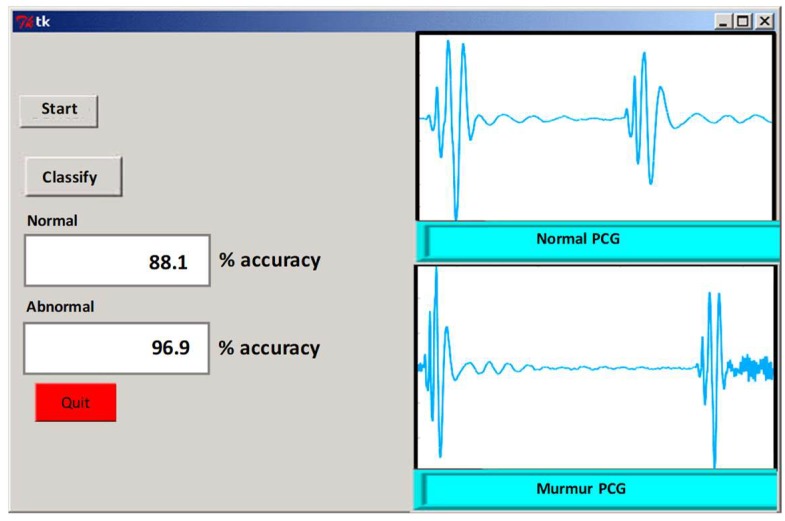
Graphical user interface for real-time HS classification using Python.

The trained model was exported to generate Matlab code, which was ported to python-based implementation for real-time classification. Python provides the benefit of multi-threaded programming to implement a separate thread to handle data acquisition from sensor subsystem and buffering data for 10 s and automatic segmenting this buffered frame into an HS (S1-S2 epoch) segment; which was used to calculate all the features in a separate thread and a dedicated classifier thread was implemented to classify the HS using the trained model. Both the recall and precision are reasonable for reliable detection and this is true for both positive and negative classification. Figure 19 shows the GUI showing the data acquisition, segmentation, and classification interface developed using python for real-time classification and tested using six normal and abnormal subjects. 

In the current study, we have proposed a system where the acquisition system is based on miniature microcontroller with low-computational capability and not suitable for machine learning model deployment. Therefore, ML algorithm was implemented in the PC-based host system. In the current design, accuracy and performance of the classification model has no influence on the battery life of the acquisition system; however, of course, the system is not very compact because of the host system. However, the overall efficiency of the implemented algorithm was highly optimized for best performance with optimum parameters: Number of iteration, distance, and number of neighbors. It can be easily ascertained that current implementation takes the advantage of parallel processing and much faster than the deep learning techniques.

## 5. Conclusions

In this study, the authors have proposed and implemented a portable heart sound capturing system for real-time heart sound anomaly detection. The digital stethoscope was designed by modifying an analog stethoscope and adding an analog front end and miniaturized microcontroller with built-in BLE for digitization and transmission. By using this device, the user can keep track of his/her heart condition on a daily basis, at low cost. A public large imbalanced dataset was used to train and test the algorithm with 27 t-domain, f-domain, and MFCC features. The best-performing algorithms in terms of classification accuracy were reported with several other statistical performance measures. Feature reduction, hyperparameter optimization, along with asymmetrical cost assignment in the training of algorithm were evaluated to obtain best performance from the algorithms. It was observed that the optimized Ensemble algorithm can outperform all the trained algorithm in classifying the test data subset. The highest score of the PhysioNet-2016 challenge competition reported overall accuracy of 86.02%, whereas the work reported has achieved a higher accuracy of 94.63% (97% abnormal and 88% normal). The classification accuracies with the cost adjustment were found to be 97% and 88% for detecting abnormal HS and normal HS, respectively. In addition, the proposed smart-stethoscope is lower in terms of power consumption and, therefore, it is expected that the device can run for several days with a 320 mAh battery. In summary, the device can contribute to excellent health monitoring and improve personal care of cardiac patients at home in a completely noninvasive manner. In the future, we would like to make the smart-stethoscope more compact in size and more professional looking with an embedded decision-making unit to classify the HS on-board. The system might be modified to send the HS data to a smart phone using BLE interface, which can classify the HS signals real-time and display results on-screen interactively, which can be a new life-saving gadget.

## Figures and Tables

**Table 1:** Extracted features.

Feature	Definition	Equation
**Mean**	Sum of all data divided by the number of entries.	$\bar{x}=\frac{\sum x}{n}$
**Median**	Value that is in the middle of ordered set of data.	Odd numbers of entries: Median = middle data entry. Even numbers of entries: Median = adding the two numbers in the middle and dividing the result by two.
**Standard Deviation**	Measure variability and consistency of the sample.	s = $\sqrt{\frac{\sum {\left(x-\bar{x}\right)}^{2}}{n-1}}$
**Percentile**	The data value at which the percent of the value in the data set are less than or equal to this value.	$25^{\mathrm{th}}=\left(\frac{25}{100}\right)n$
		$75^{\mathrm{th}}=\left(\frac{75}{100}\right)n$
**Mean Absolute Deviation**	Average distance between the mean and each data value.	MAD = $\frac{\sum_{i=1}^{n}\left|x_{i}-\bar{x}\right|}{n}$
**Inter Quartile Range**	The measure of the middle 50% of a data set.	IQR = Q_3_ – Q_1_ Q_3_: third quartile, Q_1_: first quartile, Quartile: dividing the data set into four equal portions.
**Skewness**	The measure of the lack of symmetry from the mean of the dataset.	$g1=\frac{\sum_{i=1}^{N}{\left(Y_{i}-Y\right)}^{3}/N}{s^{3}}$ Y: mean, s: the standard deviation, N: number of the data.
**Kurtosis**	The pointedness of a peak in distribution curve, in other words it’s the measure of sharpness of the peak of distribution curve.	k = $\frac{\sum_{i=1}^{N}{\left(Y_{i}-Y\right)}^{4}/N}{s^{4}}-3$ Y: mean, s: the standard deviation, N: the number of data.
**Shannon’s Entropy**	Entropy measures the degree of randomness in a set of data, higher entropy indicates a greater randomness, and lower entropy indicates a lower randomness.	H(x) = −$\sum_{i=0}^{N-1}p_{i}\log_{2}p_{i}$
**Spectral Entropy**	The normalized Shannon’s entropy that is applied to the power spectrum density of the signal.	SEN = $\frac{-\;\sum_{i=0}^{N-1}p_{k}\log_{2}p_{k}}{\log N}$ p_k_: the spectral power of the normalized frequency, N: the number of frequencies in binary
**Maximum Frequency**	The value of highest frequency in the signal spectrum	*f_max_*
**Magnitude at Fmax**	Signal magnitude at highest Frequency	X(*f_max_*)
**Ratio of signal energy**	Ratio of signal energy between *f_max_* ± Δ*f* and the whole spectrum	X (*f_max_* ± Δ*f*)$/\sum_{i=0}^{N-1}X_{i\;}\left(f\right)$
**MFCC (13 features)**	Mel-Frequency Cepstral Coefficients (MFCC): coefficients that collectively make up a Mel-Frequency Cepstral (MFC).	x = x − 0.95*[0; x (1: N-1)]; X = fft(x);

**Table 2:** Dataset observation.

	Categories	No. of Observation
**Training and Validation**	Abnormal	2505
	Normal	7907
**Testing**	Abnormal	653
	Normal	1950

**Table 3:** Performance measures of three best performing algorithms for full-feature set.

Items	Fine KNN	Weighted KNN	Ensemble Subspace Discriminant
Accuracy	94.63%	93.72	93.17%
Accuracy: Abnormal	88%,12%	85%,15%	87%, 13%
Accuracy: Normal	96.6%, 3.4%	97%,3%	95%, 5%
Error	5.37%	6.28%	6.83%
Sensitivity	96.32%	95.24%	95.67%
Specificity	89.34%	88.72%	85.49%
Precision	96.62%	96.54%	95.29%
FPR	10.66%	11.28%	14.51%
F_Score	96.46%	95.88%	95.48%
MCC	85.34%	82.7%	81.5%

**Table 4:** Performance measures of three best performing algorithms for reduced-feature set.

Items	Fine KNN	Weighted KNN	Ensemble Subspace Discriminant
Accuracy	92.36%	92.02%	92.89%
Accuracy: Abnormal	84%,16%	82%,18%	83%, 17%
Accuracy: Normal	95%, 5%	95%,5%	96%, 4%
Error	7.64%	7.98%	7.11%
Sensitivity	94.85%	94.30%	94.77%
Specificity	84.52%	84.62%	86.71%
Precision	95.08%	95.22%	95.90%
FPR	15.48%	15.38%	13.29%
F_Score	94.96%	94.76%	95.33%
MCC	79.17%	78.09%	80.42%
